# Comparison of Bovine Mammary Involution and Intramammary Infections Following Intramammary Treatment with Casein Hydrolysate and Other Conventional Treatments at Dry-Off

**DOI:** 10.3390/ani11082360

**Published:** 2021-08-10

**Authors:** Justine E. Britten, Kerry A. Rood, David J. Wilson

**Affiliations:** Animal Dairy and Veterinary Sciences Department, Utah State University, 4815 Old Main Hill, Logan, UT 84322, USA; justinebritten@gmail.com (J.E.B.); Kerry.rood@usu.edu (K.A.R.)

**Keywords:** dry cow therapy, casein hydrolysate, involution, intramammary infection, mastitis, bovine

## Abstract

**Simple Summary:**

Alternatives to antibiotic therapy for mastitis in dairy cattle are of interest to the dairy industry and society. Reduced use of antibiotics while maintaining or improving animal welfare is desirable. We studied the intramammary infusion of casein hydrolysate, alone or combined with standard dry cow treatment, at the beginning of the dry period before cows have their next calf. The effects on mammary involution and milk quality suggested that infusion of casein hydrolysate alone or combined with internal teat sealant may be an alternative to antibiotic dry cow therapy.

**Abstract:**

Alternatives to routine antibiotic treatment of dairy cattle during the dry period before their next calving are of interest. This was a preliminary study of whether intramammary infusion of casein hydrolysate, administered alone or combined with standard dry treatment, accelerated the rate of mammary involution early in the dry period. Four treatments were studied in a split udder design. One udder half was assigned a treatment, and the contralateral half was administered dry cow treatment + internal teat sealant as a control. Treatments were casein hydrolysate, casein hydrolysate + dry cow treatment, casein hydrolysate + teat sealant and casein hydrolysate + dry cow treatment + teat sealant. Cows (*n* = 16) were blocked by a number of intramammary infections per udder half (0 or 1+) and randomized to treatments. Milk production was not different between control or treated udder halves post-calving. A generalized linear mixed model tested for differences between the treatment groups in the concentration of mammary involution indicators in milk: somatic cell count, bovine lactoferrin and bovine serum albumin. At 7 days, dry udder halves treated with casein hydrolysate had higher milk concentrations of lactoferrin than those treated with casein hydrolysate + dry cow treatment, casein hydrolysate + teat sealant and control. At 10 days dry, bovine serum albumin was higher in udder halves treated with casein hydrolysate than in those treated with casein hydrolysate + dry cow treatment, casein hydrolysate + dry cow treatment + teat sealant and control. Post-calving, casein hydrolysate-treated udder halves produced 51% of total milk, unchanged from before dry-off. There were seven total intramammary infections entering the dry period, all caused by coagulase-negative staphylococci. Cure rates (3/7, 43%) were not different among all treatments and control, partly because of the small sample size. Intramammary infusion of casein hydrolysate at the end of lactation may be an alternative or possible adjunct to antibiotic dry cow therapy.

## 1. Introduction

The importance of the dry period in dairy cows has long been established within the dairy industry [1,2]. Cows may carry existing intramammary infections (IMI) into the dry period that remain present into the next lactation and are at an increased risk for acquiring new IMI immediately following cessation of milking [3,4]. Persistent and new IMI following calving have been associated with increased SCC and decreased milk production in the subsequent lactation [5]. Currently, the standard practice for mitigating IMI risk at dry-off is the blanket use of intramammary dry cow antibiotic therapy (DCT) [6,7]. Previous studies have demonstrated that blanket use of DCT (BDCT) has been effective in eliminating many existing IMI present at dry-off and preventing new IMI in the early dry period [8,9]. Additionally, the routine use of DCT has been shown to effectively lower bulk tank milk SCC [10] and nearly eradicate certain pathogens in some countries [11]. Recently, the practice of BDCT has come under international scrutiny because of concerns for antibiotic overuse [12].

Among consumers, scientists and health professionals, there is increasing concern over the widespread use of antibiotics in food-producing animals and the potential contribution to antimicrobial resistance, both in veterinary pathogens and in human medicine [13]. The data remain indeterminate on whether antibiotic use in livestock definitively causes antimicrobial resistance in humans; however, associations have been found between practices that restrict on-farm antimicrobial usage and a decrease in the prevalence of antibiotic-resistant bacteria [14]. Within the dairy industry, reducing or eliminating BDCT has become a major focus as a potential change to reduce antibiotic use. Scientific and industry interest has emerged for developing strategies for selective use of dry cow therapy (SDCT) as a possible method of reducing antimicrobial use while still maintaining the benefits of reducing the incidence rate of clinical mastitis and lowering bulk tank SCC [15,16]. Studies investigating the efficacy of SDCT have produced conflicting results [15,16]. Cows dried off without antibiotics experience higher rates of both clinical and subclinical mastitis in the following lactation, and any economic benefit gained from reducing antimicrobial use at dry-off is lost by the increase in mastitis cases [12]. There are disadvantages to both BDCT and SDCT, and a need for an alternative dry cow treatment exists within the dairy industry.

When drying off single mastitic quarters mid-lactation, casein hydrolysate intramammary infusion was associated with successful cessation of lactation in treated quarters with no signs of cow discomfort [17,18], decreased cow-level SCC during the remainder of lactation and return to milk production with decreased SCC in all previously mastitic quarters following calving [18]. Cows dried off and treated with casein hydrolysate as the only dry cow treatment exhibited more cow comfort and less mammary gland distension compared with cows dried off abruptly with no treatment [19]. When used in combination with antibiotic dry cow treatment, casein hydrolysate was associated with increased bacteriological cures and higher milk yield compared with antibiotic dry treatment alone; those authors also stated that casein hydrolysate should be studied in combination with internal teat sealants and to evaluate whether it might result in faster mammary gland involution [20]. We investigated whether intramammary infusion of casein hydrolysate would be associated with faster mammary involution in comparison to that following other dry treatments during the first 10 days of the dry period, when most of mammary involution takes place [21].

This study evaluated the effects of casein hydrolysate alone or in combination with antibiotic dry cow treatment and/or an internal teat sealant as well as a standard dry cow treatment control. The antibiotic dry cow treatment and internal teat sealant were chosen because of their widespread use in the dairy industry [22].

## 2. Materials and Methods

### 2.1. Study Animals and Permits

Study cows were housed on 6 commercial Utah dairy farms. All farms followed a twice daily milking schedule, and cows were housed on shaded dry lots that are typical of the region. Post-calving management was similar on all farms including removal of the calf from the dam soon after calving. Lactation number, days in milk (DIM) and estimated calving date were obtained from dairy records. To be eligible for the study, cows were pregnant and scheduled for dry-off within 40 to 70 days before expected parturition. Cows with nonlactating quarters or presenting with clinical mastitis were excluded. All animal handling and treatments in this study were performed in compliance with a Utah State University Institutional Animal Care and Use Committee (IACUC) approved protocol #2739.

Casein hydrolysate is not labeled for use in food animals in the U.S.; this study was conducted under conditional U.S. Food and Drug Administration (FDA) permission and followed typical guidelines for milk and meat withhold (60 d) times for commercial treatments and FDA-suggested milk withhold time (72 h) for casein hydrolysate.

### 2.2. Experimental Design

A completely randomized block design was used. All cows were blocked into two groups by number of quarters containing a subclinical IMI (either 0 or 1+). Infection status was based on culture results from a milk sample collected 4 days prior to dry-off. All aseptic milk samples for culture during the study were collected by study personnel. There were 4 treatment groups and one control: casein hydrolysate; casein hydrolysate + dry cow antibiotic (Dry-Clox, Boehringer Ingelheim, Ridgefield, CT, USA); casein hydrolysate + teat sealant (Orbeseal, Zoetis Animal Health, Parsippany, NJ, USA); casein hydrolysate + dry cow antibiotic + teat sealant; and dry cow treatment + teat sealant (control). One half of each udder, front and rear quarters combined, was randomized to a treatment group, while the other half received control, allowing every cow to serve as her own control and minimize the effect of variability between animals [23,24]. Study cows were marked with corresponding colored leg bands on both hind legs, along with an additional leg band used to identify the control side. Treatments were preassigned using a random number generator to assign which udder half would be treated versus control, followed by the treatment group assignment. Assignment of treatment sides and groups was forced only for the last cow within each block to ensure blocks were balanced with an equal number of animals per treatment group and an equal distribution of treatments and controls between right and left udder halves.

A second milk sample for culture was taken immediately prior to the time of treatment, for later evaluation of bacteriological cure. Bacteriological cure of previous IMI and new infection rates were not primary objectives of this study. However, involution is a mechanism of curing existing IMI during the dry period and an important outcome for any treatment during the dry period in dairy cattle [25,26]. Quarters which had the same organism isolated from the same quarter from both pre-treatment samples (“+, +”) were eligible for bacterial cure evaluation post-calving. Postpartum, the previously treated quarters were resampled 3 times, at 3 DIM, 8–14 DIM and 15–21 DIM.

Bacterial culture of milk samples was performed according to standard methods approved by the National Mastitis Council [27,28]. In brief, a 10 µL inoculum of milk was streaked onto washed cow blood agar and placed in a standard, non-CO_2_ incubator at 37 °C for 24 h. Plates were examined for bacterial growth at 24 and 48 h; organism identification was determined primarily by colony morphology and confirmed by secondary biochemical tests. Presence of one colony was considered positive for identification; therefore, the detection limit was 100 colony-forming units (CFU/mL). If more than 3 different types of bacteria were isolated, this was defined as contamination. However, any isolation of *Staphylococcus aureus* was defined as a valid isolate.

### 2.3. Case Definitions and Inclusion Criteria

The bacteriologic case definitions were: cure = all 3 post-calving cultures negative for any bacteria that were isolated from both pre-treatment samples; chronic (failed to cure) = any bacteria isolated from both pre-treatment samples that were also isolated from at least one post-calving culture; new IMI = any organism isolated from the first post-calving culture that was not present in either pre-treatment sample. Cows whose pre-treatment cultures resulted in no growth from one sample but growth in another (“+, −” or “−, +”) and/or different organisms isolated from each sample were ineligible for bacterial cure evaluation post-calving. All cows were eligible for new infections, including with a new pathogen if they had a different pathogen causing IMI before dry-off.

Cows meeting inclusion criteria were administered treatment at their scheduled dry-off time. All treatments were administered by study personnel. Prior to receiving treatment, all cows were bucket milked by udder half for their final milking, in order to obtain udder half milk weights and determine proportional contributions to total cow milk production. A second individual quarter milk sample was collected immediately prior to receiving treatment. Following milking and sample collection, the front and rear quarters of each udder half were infused aseptically with the preassigned treatment or control. Teats were dipped with an iodine-based germicide post-treatment, and cows were immediately moved to nonlactating housing. Individual quarter milk samples were collected again at d 2, d 4, d 7 and d 10 post-dry-off, for a total of 6 sampling dates and 24 quarter samples per cow. If teat sealant was part of the treatment, it was not administered until after the final milk sample collection at d 10 post-dry-off. In order to be minimally disruptive to the involution process, no more than 10 mL of milk was removed from each quarter per sample. 

### 2.4. Mammary Involution Markers in Milk

Collected milk samples were analyzed for the established biomarkers of involution direct microscopic SCC (DMSCC), bovine lactoferrin (bLf) and bovine serum albumin (BSA) [29]. Following parturition, milk from treated cows was withheld from the bulk tank milk for 72 h pending a negative antibiotic residue test (Delvotest-NT, Thermo Fisher, Waltham, MA, USA). At 72 h (3 DIM) post-calving, bucket milking by udder half was repeated, and individual quarter samples were collected for bacterial culture. Quarter sampling for bacterial culture was repeated once between 8 and 14 DIM and again once between 15 and 21 DIM. 

Concentrations of milk BSA and bLf were measured using the lactoferrin quantitative ELISA and bovine serum albumin quantitative ELISA kits (BioMatik, Wilmington, DE, USA). Procedures were performed according to manufacturer’s instructions. The Form 2400 FDA method was used to determine DMSCC according to the standards of the National Conference on Interstate Milk Shipments [30].

### 2.5. Casein Hydrolysate

Two batches of casein hydrolysate were prepared as previously described [18,31]. Each batch of casein hydrolysate was screened for bacterial contamination by inoculating tryptic soy broth with 1 mL of the solution, incubating for 24 h and inoculating 100 µL onto blood agar. Blood agar plates were incubated for a total of 48 h at 37 °C and read at 24 and 48 h for bacterial growth. A separate blood agar plate was plated directly with 100 µL of non-enriched casein hydrolysate and then incubated and examined in the same way. The definition of an uncontaminated batch was when no growth of any bacterial colonies was observed on either direct or enriched cultures. Protein concentration for each batch was quantified via bicinchoninic acid assay (BCA, Thomas Scientific, Swedesboro, NJ, USA), according to manufacturer’s instructions. The BCA assay is commonly used for protein quantification [32]. Final concentration of casein hydrolysate solutions was 5.6 mg/mL, for a total dose of 84 mg of hydrolyzed casein per 15 mL dose. Purity was assessed using sodium dodecyl sulfate polyacrylamide gel electrophoresis (SDS-PAGE, Thermo Fisher, Waltham, MA, USA) by comparing resulting bands to known molecular weights of hydrolyzed casein.

### 2.6. Milk Production Monitoring

All cows were bucket milked at their final milking before treatment and again at 72 h post-calving, in order to measure the proportion of total cow milk production from each udder half. This was an indication of whether any treatment was associated with increased or decreased milk production during the next lactation compared to the contralateral udder half. Bucket milking used clear, graduated 80 lb (36.3 kg) portable buckets. Two separate bucket milking units were used in order to milk each udder half. Teat cup plugs were used to seal the opposite half of the 4-claw milking unit.

### 2.7. Statistical Analysis

Statistical analyses were performed using SAS Studio (Statistical Analysis System Institute, Cary, NC, USA), using the generalized linear mixed model procedure (GLIMMIX). Milk production by udder half was compared and analyzed by Student’s t-test. The predictor variables time since dry-off and treatment were viewed as fixed effects, individual cow ID was treated as a random effect and the interaction between time and treatment was analyzed for each outcome variable. Natural log transformations were carried out for all continuous outcome variables to correct for the wide range of numerical values resulting in non-homogenous variances. Categorical outcomes of cure, chronic and new IMI were compared for significant differences between treatments using Fisher’s exact test. Differences were considered statistically significant when *p* ≤ 0.05 for all analyses.

## 3. Results

A total of 16 dairy cows, 12 Holsteins and 4 Jerseys were enrolled in this study. Cows ranged in parity from first through seventh lactations, with an average of 3.1 lactations and 337 DIM. There were no statistically significant differences between each treatment group in DIM (casein hydrolysate = 359, casein hydrolysate + dry cow treatment = 352, casein hydrolysate + teat sealant = 331, casein hydrolysate + dry cow treatment + teat sealant = 306), average lactation number (casein hydrolysate = 3.0, casein hydrolysate + dry cow treatment = 3.0, casein hydrolysate + teat sealant = 3.3, casein hydrolysate + dry cow treatment + teat sealant = 3.3) or udder half percentages of total cow milk between any of the treatment groups or the control group before treatment (all *p* ≥ 0.20, ANOVA). There were also no significant differences between pre- and post-treatment contributions of udder halves to total cow milk production within or between all treatment groups and the control (all *p* ≥ 0.06, ANOVA).

All treated cows completed the dry period and calved near their estimated due date without complication. All cows tested negative for antimicrobial residues at 72 h post-calving (Delvotest-NT). One cow died 36 h after calving for unknown reasons—mastitis was ruled out at necropsy—and was not replaced; therefore, 15 cows finished with complete datasets. Post-calving milk weight and bacterial cultures were the only data points unable to be collected from the cow that died, meaning *n* = 16 for all other outcomes.

The 15 cows (60 quarters) that survived long enough post-calving for evaluation of cures and new infections had been quarter sampled twice before treatment, at 4 days prior to treatment and again at dry-off. Twenty-seven quarters (45%) grew a bacterial organism, but only seven quarters grew the same bacterial isolate in both pre-treatment samples and were eligible for a bacterial cure evaluation. All seven intramammary infections before dry-off were caused by coagulase-negative staphylococci (CNS) isolated in pure culture. The other 20 quarters had bacterial growth in at least one of their pre-treatment samples but were ineligible for cure rate data either because one sample produced no growth or there were different isolates in the two samples. The most common isolates were CNS (*n* = 72) and non-agalactiae streptococci (*Strep* spp.) (*n* = 28) (Table 1). No samples were contaminated. There were no chronic failures of treatment defined only by a positive culture result from the third and final culture samples after calving; all cultures from 15 to 21 DIM confirmed the cures or failures already detected.

Bacterial cures during the dry period for the seven CNS cases present at dry-off were as follows: casein hydrolysate = 0/2, casein hydrolysate + dry cow treatment = 1/1, casein hydrolysate + teat sealant = 1/2, casein hydrolysate + dry cow treatment + teat sealant = N/A, control = 1/2. Therefore, the overall cure rate was 2/5 (40%) across all treatments and 1/2 (50%) for control (Table 2). Chronic (failed cure) cases were as follows: casein hydrolysate = 2/2, casein hydrolysate + dry cow treatment = 0/1, casein hydrolysate + teat sealant = 1/2, control = 1/2. New infections detected after calving were as follows: casein hydrolysate = 3/8 (two CNS, one *Trueperella pyogenes*); casein hydrolysate + dry cow treatment = 1/8 (*Strep* spp.); casein hydrolysate + teat sealant = 1/6 (CNS); casein hydrolysate + dry cow treatment + teat sealant 1/8 (*Strep* spp.); and control = 8/30 (four CNS, three *Strep* spp., one *Prototheca* spp.). Therefore, overall new infection rates were 6/30 (20%) of quarters across all treatments and 8/30 (27%) of control quarters (Table 2). The cure rate was not significantly different between treatment groups or control (Fisher’s exact test, all *p* ≥ 0.33). There were also no significant differences in new infection rates during the dry period (Fisher’s exact test, all *p* ≥ 0.65).

**Figure 1:**
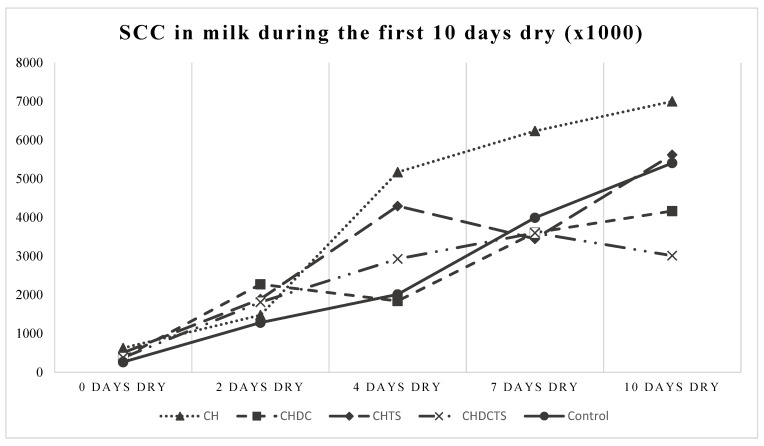
SCC (thousands/mL) by days dry and dry treatment group; no significant differences between treatments found. **CH**= casein hydrolysate; **CHDC** = casein hydrolysate + dry cow treatment; **CHTS** = casein hydrolysate + teat sealant; **CHDCTS** = casein hydrolysate + dry cow treatment + teat sealant; **Control** = dry cow treatment + teat sealant.

**Figure 2:**
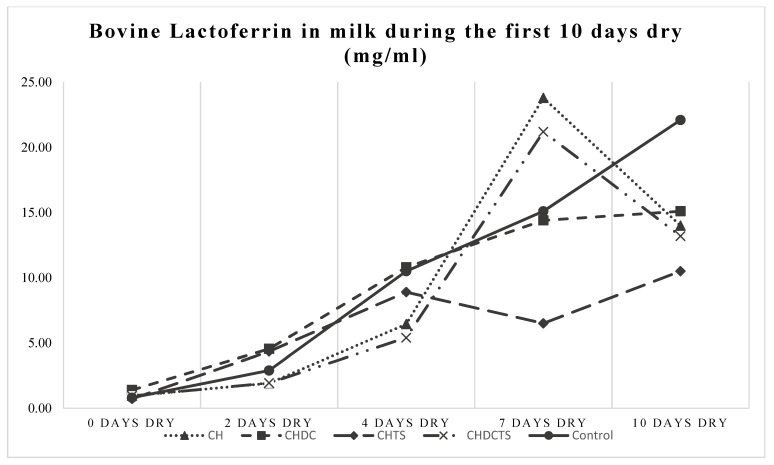
BLf by days dry and dry treatment group; CH treatment group different from CHDC, CHTS and control at 7 days dry (*p* < 0.05, GLIMMIX). **CH** = casein hydrolysate; **CHDC** = casein hydrolysate + dry cow treatment; **CHTS** = casein hydrolysate + teat sealant; **CHDCTS** = casein hydrolysate + dry cow treatment + teat sealant; **Control** = dry cow treatment + teat sealant.

**Figure 3:**
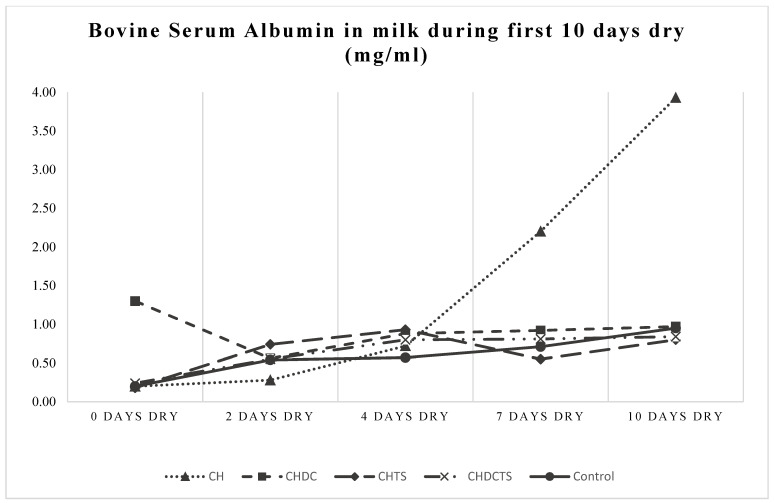
BSA by days dry and treatment group; CH cows differed from CHDC, CHDCTS and control at 10 days dry (*p* < 0.05, GLIMMIX). **CH** = casein hydrolysate; **CHDC** = casein hydrolysate + dry cow treatment; **CHTS** = casein hydrolysate + teat sealant; **CHDCTS** = casein hydrolysate + dry cow treatment + teat sealant; **Control** = dry cow treatment + teat sealant.

All measured outcome variables (indicators of involution) increased significantly from the day of drying-off (d 0) to d 10 (*p* < 0.0001, GLIMMIX), which is to be expected as a natural process of involution [29,33,34] (Figure 1, Figure 2 and Figure 3). However, no significant differences were found in DMSCC between treatment groups (*p* > 0.30), nor was there a time × treatment effect on DMSCC (*p* > 0.61) (Table 3, Figure 1). Only bLf and BSA showed a significant time × treatment interaction (*p* = 0.01, GLIMMIX) (Table 3, Figure 2 and Figure 3). Cows in the casein hydrolysate group had a higher concentration of bLf at d 7 than cows in the casein hydrolysate + dry cow treatment and casein hydrolysate + teat sealant treatment groups and control (all *p* ≤ 0.02, GLIMMIX) (Table 3, Figure 2). At d 10, cows dry treated with casein hydrolysate had a higher concentration of BSA than casein hydrolysate + dry cow treatment, casein hydrolysate + dry cow treatment + teat sealant and control cows (all *p* ≤ 0.04, GLIMMIX) (Table 3, Figure 3).

## 4. Discussion

This is the first study conducted in North America to investigate the efficacy of using intramammary casein hydrolysate to supplement or possibly replace standard dry cow therapy. The results from this study demonstrate an association between the use of casein hydrolysate and a faster increase in the milk concentration of some markers of mammary involution in comparison with standard industry dry treatment during the early dry period. Involuted bovine mammary glands are quite resistant to new IMI [33,35], which suggests that intramammary use of casein hydrolysate may be beneficial to udder health at the time of dry-off, with or without the addition of an antibiotic. All markers of involution increased from the time of dry-off and treatment to the final sampling 10 days later regardless of treatment, consistent with previously published studies [29,34].

Increases in the concentration of bovine lactoferrin and bovine serum albumin in milk have been used as indicators of bovine mammary involution previously [29,33,34,36]. Besides being an indicator of mammary involution, bovine lactoferrin plays an immunologically important role during mammary involution, by binding available iron and making it unavailable for iron-dependent bacteria to grow [37,38]. It has been reported that lactoferrin presents a robust defense against IMI [36,37,38]. One week after drying off, the concentration of lactoferrin was higher in cows that were treated with casein hydrolysate compared with the controls and all but one other treatment. Therefore, it is possible these animals experienced udder health benefits, both because of faster involution as indicated by a higher concentration of lactoferrin and possibly because of its immunologic effects as well [37]. This is an interesting and valuable outcome when considering the use of alternatives to antimicrobial dry treatment.

It was observed that the cure rate during the dry period of IMI that were present pre-treatment and the rate of new IMI during the dry period were not different between any of the treatment groups, including casein hydrolysate, antibiotic dry treatment or teat sealant in combinations. However, the logistics of the project necessitated a relatively small sample size of 16 cows, which contributed to this lack of statistical significance because of the small numbers of IMI. This is a preliminary study, and future studies with a larger sample size are needed.

## 5. Conclusions

Cows experienced no mammary quarters with proportional milk production loss following any of the dry treatments containing casein hydrolysate. Restricting blanket use of dry cow therapy in the U.S. dairy industry may become a reality in the future, and the results of this study show that intramammary use of casein hydrolysate was associated with faster increases in some measures of bovine mammary involution during the early dry period. The use of casein hydrolysate alone or combined with internal teat sealant may be an alternative to antibiotic dry cow therapy.

## Figures and Tables

**Table 1:** Bacteria isolated from all milk cultures before dry-off and after calving.

	Time When Sampled and Culture Results (Number of Qtrs. Positive)					
Bacteria	D-4 ^¥^	D0 ^£^	3 DIM ^∞^	8–14 DIM	15–21 DIM	New IMI ^γ^
CNS ^§^	12	15	17	15	13	7
*Strep* spp. ^†^	5	1	6	8	8	5
*Staph aureus*	1	0	0	0	0	0
*Pseudomonas* spp.	0	4	0	1	1	0
*E. coli*	0	5	0	0	0	0
*Prototheca* sp.	0	0	1	1	1	1
*Proteus* spp.	0	0	0	1	0	0
*Trueperella pyogenes*	0	0	1	1	1	1
No Growth	48	42	38	41	41	0
**Total**	66	67	63	68	65	14

^¥^ D-4 = 4 days before dry-off and dry treatment; ^£^ D0 = immediately before dry-off and dry treatment; ^∞^ DIM = days in milk; ^§^ CNS = coagulase-negative staphylococci (includes 7 on D-4 and D0 in same quarters that defined pre-treatment intramammary infection (IMI) in Table 2); ^†^ *Strep* spp. = non-agalactiae streptococci; ^γ^ New IMI = Any organism isolated from the first post-calving culture that was not present in either pre-treatment sample. These are the 14 new IMI shown in Table 2.

**Table 2:** Bacteriological cures and chronic cases of intramammary infections during the dry period by treatment group.

	Pre-Treatment Culture Results (Number of Qtrs. Infected)			Post-Treatment Culture Results		
Treatment Group	IMI ^¥^	Mixed ^£^	NG ^∞^	Cure ^§^	Chronic ^†^	New IMI ^γ^
Casein hydrolysate	2	5	1	^a^ 0% (0/2)	^b^ 100% (2/2)	^c^ 38% (3/8)
Casein hydrolysate + dry cow treatment	1	1	6	^a^ 100% (1/1)	^b^ 0% (0/1)	^c^ 13% (1/8)
Casein hydrolysate + teat sealant	2	1	3	^a^ 50% (1/2)	^b^ 50% (1/2)	^c^ 17% (1/6)^1^
Casein hydrolysate + dry cow treatment + teat sealant	0	1	7	N/A	N/A	^c^ 13% (1/8)
Control	2	12	16	^a^ 50% (1/2)	^b^ 50% (1/2)	^c^ 27% (8/30) ^1^
**Total**	7	20	33	43% (3/7)	57% (4/7)	23% (14/60)

^¥^ IMI = Intramammary infection; same mastitis organism isolated from both pre-treatment samples; IMI are eligible to be either cure or chronic following dry cow treatment and subsequent calving. All 7 IMI were caused by coagulase-negative staphylococci isolated in pure culture. ^£^ Mixed = different mastitis organisms isolated from pre-treatment cultures (all isolates shown in Table 1); ^∞^ NG = no growth in either pre-treatment sample; ^§^ Cure = all 3 post-treatment cultures negative for pre-treatment IMI organism; ^†^ Chronic = at least 1 post-treatment culture positive for pre-treatment IMI organism; ^γ^ New IMI = any organism isolated from the first post-calving culture that was not present in either pre-treatment sample (all isolates shown in Table 1); ^1^ One cow died before post-treatment cultures could be taken; values within a column with the same superscript are not significantly different (*p* > 0.05).

**Table 3:** Means of milk concentrations (mg/mL) of indicators of mammary involution by days dry and treatment group.

		Casein Hydrolysate	Casein Hydrolysate + Dry Cow Treatment	Casein Hydrolysate + Teat Sealant	Casein Hydrolysate + Dry Cow Treatment + Teat Sealant	Control
BSA						
0 days dry	$\bar{x}$	^a^ 0.20 (*n* = 8)	^a^ 1.30 (*n* = 8)	^a^ 0.18 (*n* = 8)	^a^ 0.24 (*n* = 8)	^a^ 0.20 (*n* = 32)
	SD	0.22	2.89	0.16	0.21	0.24
2 days dry	$\bar{x}$	^a^ 0.28 (*n* = 8)	^a^ 0.56 (*n* = 8)	^a^ 0.74 (*n* = 8)	^a^ 0.55 (*n* = 8)	^a^ 0.54 (*n* = 32)
	SD	0.01	0.71	0.80	0.45	0.53
4 days dry	$\bar{x}$	^a^ 0.72 (*n* = 8)	^a^ 0.88 (*n* = 8)	^a^ 0.93 (*n* = 8)	^a^ 0.80 (*n* = 8)	^a^ 0.57 (*n* = 32)
	SD	0.53	0.63	0.85	0.57	0.48
7 days dry	$\bar{x}$	^a^ 2.20 (*n* = 8)	^a^ 0.92 (*n* = 8)	^a^ 0.55 (*n* = 8)	^a^ 0.81 (*n* = 8)	^a^ 0.71 (*n* = 32)
	SD	3.02	0.67	0.43	0.72	0.64
10 days dry	$\bar{x}$	^a^ 3.93 (*n* = 8)	^ab^ 0.97 (*n* = 8)	^a^ 0.80 (*n* = 8)	^ab^ 0.84 (*n* = 8)	^ab^ 0.95 (*n* = 32)
	SD	5.40	0.53	0.57	0.65	0.66
bLf						
0 days dry	$\bar{x}$	^c^ 0.96 (*n* = 8)	^c^ 1.40 (*n* = 8)	^c^ 0.72 (*n* = 8)	^c^ 0.95 (*n* = 8)	^c^ 0.81 (*n* = 32)
	SD	0.54	1.24	0.26	1.03	0.89
2 days dry	$\bar{x}$	^c^ 1.92 (*n* = 8)	^c^ 4.55 (*n* = 8)	^c^ 4.35 (*n* = 8)	^c^ 1.94 (*n* = 8)	^c^ 2.90 (*n* = 32)
	SD	1.02	5.27	6.89	1.19	3.40
4 days dry	$\bar{x}$	^c^ 6.44 (*n* = 8)	^c^ 10.8 (*n* = 8)	^c^ 8.90 (*n* = 8)	^c^ 5.40 (*n* = 8)	^c^ 10.5 (*n* = 32)
	SD	8.80	8.89	3.84	4.43	12.44
7 days dry	$\bar{x}$	^c^ 23.8 (*n* = 8)	^cd^ 14.4 (*n* = 8)	^cd^ 6.50 (*n* = 8)	^c^ 21.2 (*n* = 8)	^cd^ 15.1 (*n* = 32)
	SD	13.35	14.12	3.76	15.68	15.02
10 days dry	$\bar{x}$	^e^ 14.0 (*n* = 8)	^e^ 15.1 (*n* = 8)	^e^ 10.5 (*n* = 8)	^e^ 13.2 (*n* = 8)	^e^ 22.1 (*n* = 32)
	SD	8.96	17.09	8.00	9.85	29.05
DMSCC (×1000)						
0 days dry	$\bar{x}$	^e^ 628 (*n* = 8)	^e^ 380 (*n* = 8)	^e^ 505 (*n* = 8)	^e^ 378 (*n* = 8)	^e^ 264 (*n* = 32)
	SD	607	282	945	341	215
2 days dry	$\bar{x}$	^e^ 1476 (*n* = 8)	^e^ 2274 (*n* = 8)	^e^ 1892 (*n* = 8)	^e^ 1811 (*n* = 8)	^e^ 1280 (*n* = 32)
	SD	1861	2955	2905	1621	1284
4 days dry	$\bar{x}$	^e^ 5171 (*n* = 8)	^e^ 1841 (*n* = 8)	^e^ 4296 (*n* = 8)	^e^ 2929 (*n* = 8)	^e^ 2012 (*n* = 32)
	SD	5589	1269	4887	2542	1709
7 days dry	$\bar{x}$	^e^ 6236 (*n* = 8)	^e^ 3607 (*n* = 8)	^e^ 3449 (*n* = 8)	^e^ 3601 (*n* = 8)	^e^ 3993 (*n* = 32)
	SD	3470	2886	4323	3412	3643
10 days dry	$\bar{x}$	^e^ 7001 (*n* = 8)	^e^ 4167 (*n* = 8)	^e^ 5622 (*n* = 8)	^e^ 3014 (*n* = 8)	^e^ 5407 (*n* = 32)
	SD	1700	2021	2145	2154	3078

^a–e^ Means within a row with different superscripts differ at *p* < 0.05. **BSA** = bovine serum albumin; **bLf** = bovine lactoferrin; **DMSCC** = direct microscopic somatic cell count. $\bar{x}$ = mean; SD = standard deviation.

## Data Availability

Not applicable.
